# P-1598. Evaluating the Utility of MRSA Nares Testing as an Antimicrobial Stewardship Tool

**DOI:** 10.1093/ofid/ofae631.1765

**Published:** 2025-01-29

**Authors:** Faiza Morado, Neha Nanda, Rebecca Lee

**Affiliations:** Keck Medicine of USC, Pasadena, California; USC, los angeles, CA; Keck Hospital of University of Southern California, Los Angeles, California

## Abstract

**Background:**

A common challenge for antimicrobial stewardship programs is limiting vancomycin usage as overuse is linked with significant clinical consequences such as nosocomial infections. While Methicillin-resistant *Staphylococcus aureus* (MRSA) nasal screens are widely used to rule out MRSA in lower respiratory tract infections, studies evaluating its use in non-pneumonic processes are limited. The purpose of this study was to evaluate the utility of MRSA nasal screens by estimating its predictive value for various clinical cultures.

**Figure:**
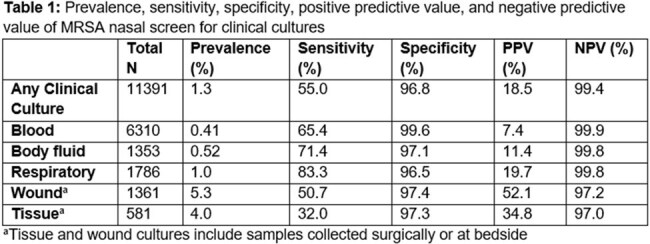

**Methods:**

This retrospective cohort study was conducted at an academic medical hospital from October 1, 2021 to January 31, 2024. Patients with an MRSA nasal swab collected within 48 hours of admission and a clinical culture within 14 days of the nasal swab were included. Chromogenic media was used for detection of MRSA colonization. Clinical cultures were defined as blood, body fluid, respiratory, wound, and tissue cultures. Patients with a history of an MRSA infection within prior 90 days or received 10 or more days of an anti-MRSA antibiotic prior to nasal swab collection were excluded. Microbiological and clinical data were collected for included patients. The primary endpoint was to determine the sensitivity, specificity, positive predictive value (PPV) and negative predictive value (NPV) for a positive MRSA clinical culture.

**Results:**

A total of 11391 clinical cultures collected from various anatomical sites were included in this study. The distribution of clinical cultures were as follows: 55.4% blood, 11.9% wound, 11.9% body fluid, 15.7% respiratory, and 5.1% tissue. The total MRSA prevalence for any clinical culture was 1.3%. The sensitivity, specificity, PPV, and NPV for all clinical cultures were 55%, 96.8%, 18.5%, and 99.4%, respectively. Results for each clinical culture site are listed in table 1.

**Conclusion:**

The results of an MRSA nasal screen via chromogenic media had a high NPV for MRSA growth in blood, body fluid, respiratory, wound, and tissue cultures up to 14 days after MRSA nasal swab collection.

**Disclosures:**

**All Authors**: No reported disclosures

